# Efficacy of transversalis fascia plane block as a novel ındication for varicocelectomy surgery: prospective randomized controlled study

**DOI:** 10.1186/s12871-023-02009-z

**Published:** 2023-02-07

**Authors:** Erkan Cem Celik, Isa Ozbey, Muhammed Enes Aydin, Ahmet Murat Yayik, Elif Oral Ahiskalioglu, Ibrahim Hakki Tor, Ali Ahiskalioglu

**Affiliations:** 1 Medical Faculty of Atatürk University, Deparment of Anesthesiology and Reanimation, Erzurum, Turkey; 2 Clinical Research, Development and Design Application and Research Center, Ataturk University School of Medicine, Erzurum, Turkey; 3 Medical Faculty of Atatürk University, Department of Urology, Erzurum, Turkey; 4 Deparment of Anesthesiology and Reanimation, Medical Faculty of University of Health Sciences, Erzurum Regional Training and Research Hospital, Erzurum, Turkey

**Keywords:** Varicocelectomy, Pain, Analgesia, Infiltration, Postoperative, Anesthesia

## Abstract

**Background:**

Varicocele occurs as a result of dilatation of the pampiniform plexus in the spermatic veins. In this study, our primary aim was to evaluate the effect of Transversalis Fascia Plane Block (TFPB) on pain scores in the postoperative period in patients undergoing varicocelectomy surgery, and our secondary aim was to evaluate the effect of TFPB on analgesic consumption.

**Methods:**

The study was initiated following local ethics committee approval, and sixty ASA I-II patients > 18y scheduled to undergo varicocelectomy and who consented to participation were enrolled. Before the procedure, the patients were randomly assigned two groups: Transversalis Fascia Plan block group (Group TFPB) or surgical incision site infiltration group (Group I).All surgeries were carried out under general anesthesia, and microsurgery using the subinguinal approach. After surgical suturing, TFPB and local infiltration blocks were applied prior to termination of anesthesia.For each block, 20 mL of 0.25% bupivacaine was utilized. Patients' demographic information, passive and active VAS ratings after surgery, usage of non steroidal anti-inflammatory medications and rescue analgesia, and the requirement for rescue analgesia, were recorded.

**Results:**

A total of 60 patients were included in the study. In terms of demographic data, there was no difference between the groups. At all hours, there was a statistically significant decrease in favor of Group TFPB in terms of active and passive VAS scores (*p* < 0.001), non steroidal anti-inflammatory analgesic use (*p* < 0.05), and tramadol requirement (*p* < 0.001).

**Conclusion:**

This study has shown that TFPB can provide a more effective analgesia when compared to surgical site infiltration.

## Background

Varicocele is caused by the dilatation of the pampiniform plexus in the spermatic veins and is observed in 15–20% of post-pubertal men [1]. Although varicocele is one of the most common causes of primary infertility, its presence is also a significant finding of other pathologies including renal cell carcinoma, which causes obstruction of the inferior vena cava (IVC), IVC thrombus, and renal vein thrombosis. The majority of patients with varicocele do not require surgery, although a sizable percentage does [2].

Many techniques exist for the surgical treatment of varicocele including, open techniques (retroperitoneal, inguinal and scrotal approaches), surgical ligation, laparoscopic approach and inguinal and subinguinal microsurgery [3, 4]. Low recurrence rates are noteworthy in varicocelectomy surgeries where microsurgical methods are used. Additionally, subinguinal varicocelectomy approach reduces the postoperative pain scores [5].

General anesthesia, central anesthetic interventions and local anesthesia can be used for analgesia and anesthesia in varicocelectomy surgery, which includes dissection of the skin and subcutaneous tissues as well as the ligation of the spermatic vein [3]. Although varicocele surgery is described as mild in terms of pain, plane blocks have been utilized extensively for the multimodal management of postoperative analgesia in lower abdominal, iliac crest bone harvesting and inguinal area procedures [6–8]. Use of plane blocks such as quadratus lumborum block, erector spinae plane block, transversus abdominis plane block, and transversalis fascia plane block have frequently been used as part of multimodal analgesia and are reported to be effective in providing low pain scores and analgesic consumption, particularly in inguinal, lower abdomen and hip surgeries [9–11].

Transversalis fascia plane block (TFPB) aims to provide analgesia for invasive procedures of the inguinal and subinguinal areas by blocking the subcostal (T12), ilioinguinal (L1) and iliohypogastric (T12-L1) nerves. Several studies have reported TFPB as the analgesic method of choice for procedures involving the T12-L1 dermatome region, including iliac bone graft harvesting, cesarean section, and inguinal hernia repair [7, 8, 10, 12].

Although varicocele surgery is described as mild in terms of pain, the fact that the pain associated with surgical incision can be removed entirely with TFPB. In this study, the primary objective was to assess the effect of TFPB on postoperative pain scores in patients undergoing unilateral varicocelectomy surgery, and the secondary objective was to assess the effect of TFPB on consumption of analgesics.

## Methods

### Study design

This randomized, controlled study was registered with clinicaltrials.gov (ref: NCT05172882, first registration date was 29/12/2021) following local institutional review board of the Ethical Committee for Clinical Research of the Medical Faculty of Ataturk University, Erzurum, Turkey. The trial was conducted between November 2021 and April 2022. The study included ASA (American Society of Anesthesiology) Class I-II patients aged > 18y, and informed written consent was obtained. Patients with known allergic reactions to any of the study's drugs (bupivacaine and other analgesic drugs), severe systemic disease (chronic renal failure, liver insufficiency, chronic obstructive pulmonary disease, etc.), all chronic pains or history of analgesic use for long-duration or coagulopathy, patients with cognitive problems or unable to understand study protocol and communication difficulties, BMI greater than or equal to 30 and patients who refused participation in the study were excluded.

Before the procedure, the patients were randomly assigned to one of two groups: TFPB group (Group TFPB) and surgical incision site infiltration group (group I) using a computer-generated randomization table. The patient study numbers and study group information were organized in two columns. The third column was created using the "RAND" function to randomly select the data in the first two columns. In this manner, patients were assigned to study groups in this column randomly. Postoperative pain evaluation was performed by a researcher who was not involved in the perioperative period of the study.

### Management of surgery, anesthesia and TFP block

Patients were placed in the supine position. Following patient monitoring with electrocardiography (ECG), non-invasive blood pressure (NIBP) and peripheral oxygen saturation (SpO_2_), patients were placed under general anesthesia with a laryngeal mask, as is routine for patients undergoing varicocelectomy in our clinic. All patients received 2.5 mg/kg propofol and 1.5 mcg/kg fentanyl for induction, prior to insertion of a laryngeal mask. Anesthesia was maintained using 1 MAC sevoflurane and 50% O_2_-50% N_2_O air mixture. The surgical team then began the procedure. All surgeries were carried out using a Leica® M525 F40 surgical microscope with magnification ranges between 10-15x. For surgery, a 2–2.5 cm long subinguinal incision was performed. The spermatic cord was then dissected. For cord retraction, a penrose drain was carefully placed from the posterior side of the chord. The testicular artery, vasal vein, and lymphatic arteries were all preserved while all dilated vessels were ligated. The veins were examined before closure, and the wound lips were sutured with absorbable threads.

**Fig. 1:**
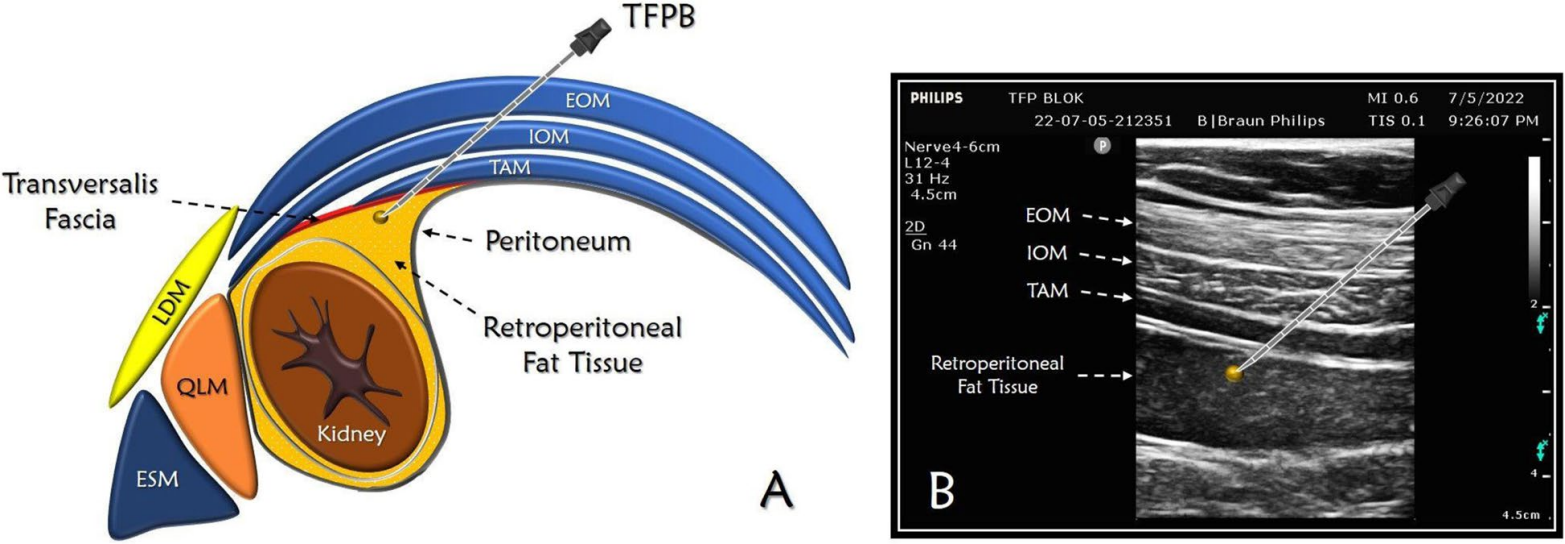
Anatomic illustration and ultrasonographic image of transversalis fascia plane block intervention. **A**; Anatomic illustration of TFPB and other anatomical structures. **B**; Sonographic image of TFPB. EOM; external oblique muscle, IOM; internal oblique muscle, TAM; transversus abdominis muscle, LDM: latissimus dorsi muscle, QLM: quadratus lumborum muscle, ESM: erector spinae muscle

Postoperatively, TFPB and infiltration analgesia were performed by experienced anesthesiologists or urologists, respectively. TFPB was performed under sterile conditions using a linear ultrasound probe (Esaote®, Mylab 5, Florence, Italy) and a 80 mm block needle (B.Braun®, Melsungen, Germany) was used to administer local anesthesic below the transversus abdominis muscle into the perirenal adipose tissue, taking into account the borders of the abdominal cavity. Local anesthetic used in both Group TFPB and group I consisted of 20 ml of 0.25% bupivacaine. In group I, the local anesthetic was administered into the cutaneous and subcutaneous tissues. (Fig. 1).

In both groups, interventions were performed at the end of the surgery before anesthesia was terminated. Patients who required additional surgery apart from varicocelectomy or intubation due to airway management issues were excluded from the study.

### Outcomes and postoperative analgesic management

Patients' demographic data, such as age, weight, and height, ASA classification, duration of anesthesia and surgery, passive (at rest) and active (from supine to 45° sitting position) postoperative VAS scores at 1, 2, 4, 8, 12, and 24 h after surgery (VAS 0 = no pain, VAS 10 = worst pain imaginable) were recorded.

The patients were administered 1000 mg paracetamol iv around 30 min before the procedure ended. Following the procedure, the patients were observed in the recovery unit for follow-up. In the postoperative phase, patients in both groups were administered 1000 mg of paracetamol at 6-h intervals after first dose is given. When the VAS score was determined to be ≥ 4, 1 mg/kg tramadol was administered as a rescue opioid analgesic. IV 50 mg dexketoprofen was administered in the ward when the VAS score was greater than 2.

### Sample size and statistical analyze

Data from a preliminary study taking into account average VAS score at 2^nd^ postoperative hour (Group TFPB: 2.14 ± 0.85 (n: 5) vs Group I: 3.21 ± 1.24 (n: 5)) was used to calculate a minimum sample size of 23 participants per group (type 1 error = 0.05, type 2 error = 0.10 and power = 0.95). The final decision was taken to include 30 patients per group, taking into account possible losses to follow-up.

Statistical analysis was performed using statistical program (SPSS Statistics version 20.0-IBM, Armonk, NY,USA). Data normality was evaluated using the Kolmogorov–Smirnov test. Mann Whitney-U test was used for continuous data that did not show normal distribution. Independent t-test was used for normally distrubuted data. Fisher Exact test was used to evaluate categorical data. *p* < 0.05 were considered to be statistically significant.

## Results

**Fig. 2:**
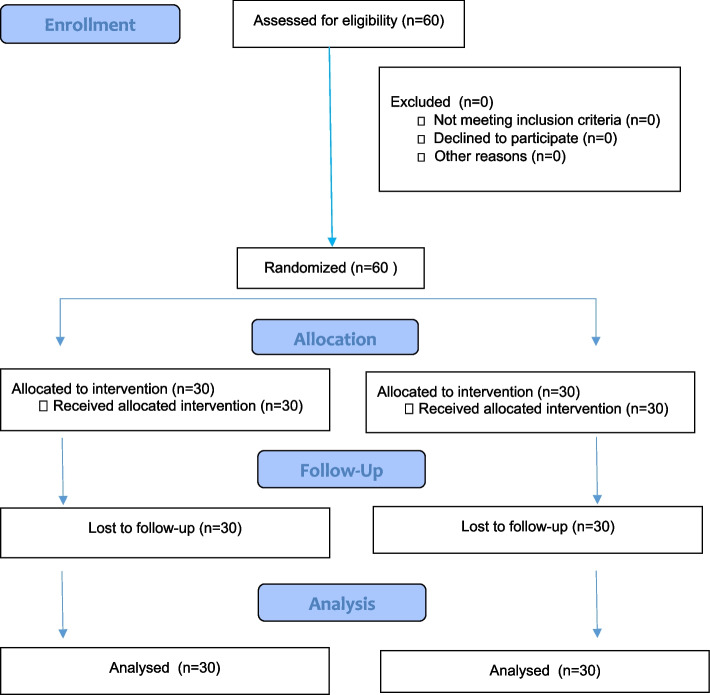
Consort Diagram of the study

A total of 60 patients were included in the study (Fig. 2). No patients were excluded. Demographic data such as age, weight, height, ASA classsification, duration of anesthesia and surgery were similar between both groups (Table 1).

**Table 1:** Demographic data and comparison of operative procedures between groups

	**Group TFP (n:30)**	**Group I (n:30)**	**p**^α^
Age (years)	28 (25–32)	30 (26–32)	0.588
Weight (kg)	76 (71–85)	75 (71–82)	0.519
ASA (I-II)	30/0	30/0	N
Height (cm)	176.5 (170–182)	172.5 (169–178)	0.310
Duration of Anesthesia (min)	65 (60–75)	65 (60–70)	0.633
Duration of Surgery (min)	50 (50–60)	55 (50–60)	0.517

Values are expressed median (%25–75) or number

*ASA* American Society of Anesthesiologist, kg; kilogram, cm; centimeter, min; minutes

^α ^*p* > 0,05 Mann–Whitney U

**Fig. 3:**
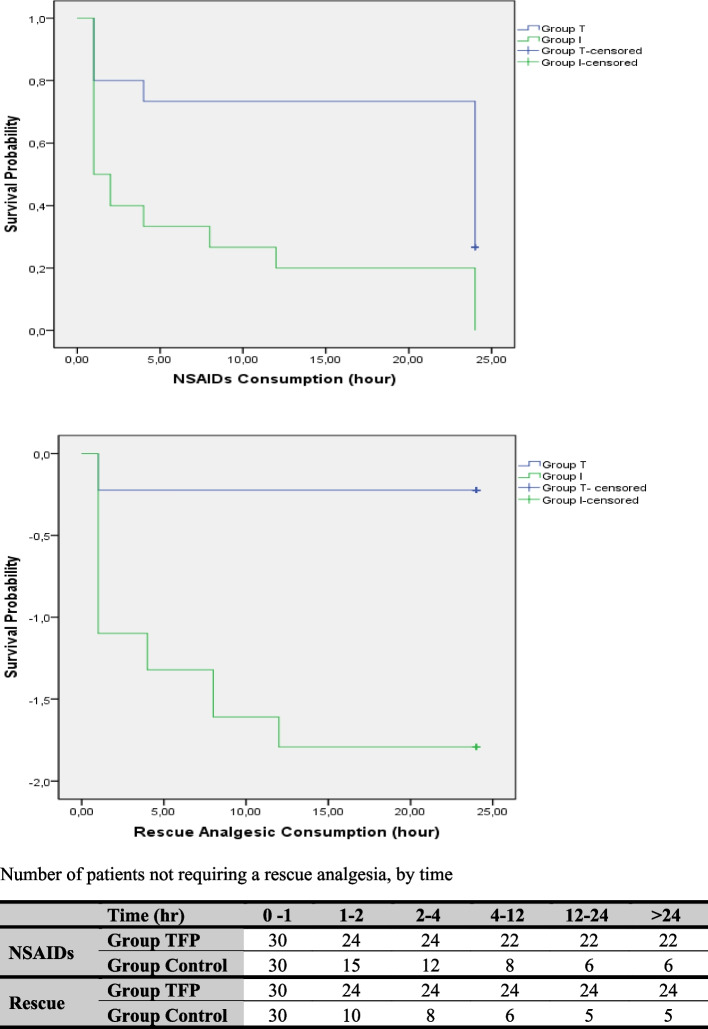
Kaplan–Meier survival curves for non steroidal anti-inflammatory drugs and rescue analgesics of the patients during first day. (log rank *p* < 0.05)

When active and passive VAS scores at all time points were compared, VAS scores in Group TFPB were statistically significantly lower when compared to group I (*p* < 0.001). Consumption of non-steroidal anti-inflammatory drugs was also lower in Group TFPB than Group I (8/30vs. 25/30, respectively, *p* < 0.05). Number of patients requiring for rescue analgesia (tramadol) was also statistically significantly lower in Group TFPB than Group I (6/30 vs. 25/30, respectively, *p* < 0.001). (Table 2 and Fig. 3). No surgery or anesthesia related complication (hemorrhage, respiratory depression, nausea, vomiting etc.) was observed.

**Table 2:** Comparison of the VAS score assessment between groups at different time points

	**Group TFP (n:30)**	**Group I (n:30)**	**p**
Passive 1^st^	2 (1–3)	3 (3–5)	< 0.001^α^
Passive 2^nd^	1.5 (1–2)	3 (2–3)	< 0.001^α^
Passive 4^th^	1 (1–2)	3 (2–3)	< 0.001^α^
Passive 8^th^	1 (0–2)	2.5 (2–4)	< 0.001^α^
Passive 12^th^	1 (0–2)	2 (2–4)	< 0.001^α^
Passive 24^th^	1 (0–1)	2 (1–3)	< 0.001^α^
Active 1^st^	3 (2–4)	5 (4–7)	< 0.001^α^
Active 2^nd^	2 (1–3)	4 (3–4)	< 0.001^α^
Active 4^th^	2 (1–3)	3 (2–4)	< 0.001^α^
Active 8^th^	2 (1–3)	3 (2–4)	< 0.001^α^
Active 12^th^	2 (1–2)	3 (2–4)	< 0.001^α^
Active 24^th^	1.5 (1–2)	2.5 (2–3)	< 0.001^α^
NSAID consumption in ward (Y/N)	8/22	25/5	0.005 ^β^
Tramadol Consumption (Y/N)	6/24	25/5	< 0.001 ^γ^

Values are expressed median (%25–75) or number

*NSAID* non-steroid anti-inflammatory analgesig drugs

^α ^*p* < 0,05 Mann–Whitney U test

^β ^*p* < 0,05 Fisher’s Exact test

^γ^ *p* < 0.001 Fisher’s Exact test

## Discussion

To the best of our knowledge, this is the first study to evaluate the effectiveness of TFBP block for postoperative analgesia after varicocelectomy surgery. Our study has shown that when compared to local anesthetic infiltration of the surgical site, TFBP block decreases pain scores, non-steroid analgesic requirement and rescue analgesia consumption.

Subinguinal incisions are often preferred in microsurgical varicocelectomy because the spermatic cord is more superficial and the anatomy is simpler. As a result, there is far less dissection, manipulation, and pain. This leads to less discomfort, less need for analgesics and earlier mobilization. As a result, microsurgical varicocelectomy with accelerated recovery after surgery (ERAS) is performed on a regular basis in our clinic. Furthermore, as effective postoperative analgesia is essential for ERAS, TFPB is commonly used in patients undergoing varicocelectomy in our clinic, as part of multimodal analgesia.

The somatic innervation of the subinguinal incision used in varicocelectomy surgery is through the subcostal nerve (T12-L1), ilioinguinal nerve (L1) and the iliohypogastric nerve (T12-L1) [9]. Blockage of these nerves is possible through several different analgesic/anesthetic methods such as central nerve blocks (spinal anesthesia, epidural anesthesia or combined spina-epidural anesthesia), peripheral nerve blocks (transversus abdominis plane (TAP) blocks, ilioinguinal block, quadratus lumborum plane block (QLB), TFPB etc.) and skin infiltration [13]. Central interventions are generally not preferred in short lasting surgical procedures.

When the abdominal muscles are evaluated, the rectus muscles are near the linea alba. Traveling to the lateral part of the abdomen, there are three muscles: superficial to deep, the external oblique, internal oblique, and transversus abdominis muscles. TAP block is performed between the internal oblique and transversus abdominis muscles. This interfascial area is vast compared with the perirenal fat tissue compartment. The transversalis fascia is a thin fibrous membrane structure located behind the transversus abdominis muscle that limits perirenal fat tissue. The TFPB can be used to block the subcostal, ilioinguinal, and iliohypogastric nerves while they are running through the perirenal adipose tissue [14]. TFPB is not cover the visceral peritoneum, but TFPB covers the parietal peritoneum part of T12-L1. TFPB is easier to apply compared to TAP, because it is applied to the perinephric adipose tissue without interfering with the fasciae and is more posterior to the abdominal cavity with lower risk of inadvertent puncture of the abdominal cavity. The prospect of a difficult attempt in a supine position, especially in QLB II and III, makes TFPB a significant alternative [10].

TFBP was first reported in inguinal hernia surgery, appendectomy and in bone harvesting from the iliac crest. Thereafter, TFPB applications were reported for similar surgeries where somatic innervation was also from T12 and L1. TFBP application was compared to spinal anesthesia for postoperative analgesia in cesarean section and those undergoing TFBP were found to have lower morphine consumption [10]. In a separate study of patients undergoing cesarean section under general anesthesia, visual analog scale (VAS) and tramadol consumption were found to be lower in patients undergoing TFPB block for postoperative analgesia, compared to a control group [12]. Separately, TFPB block was shown to offer similar effectiveness, ease of application and patient satisfaction when compared to TAP block in patients undergoing lower abdominal surgery, such as inguinal hernia surgery. Furthermore, case reports by Scimia et al. and Ahiskalioglu et al. and a randomized controlled study by Serifsoy et al. demonstrated that TFPB could be an appropriate choice in patients undergoing inguinal hernia surgery [12, 15, 16].

Increased success and decreased complication rates, such as unintended vascular access, pneumothorax, nerve injuries, etc., have been observed with peripheral nerve blocks performed under ultrasound guidance. Especially in cases where neurostimulation cannot be used, such as plane blocks, ultrasound can further increase the success of the block.

This study has some limitations. Firstly; TFPB block was administered during general anesthesia after the end of surgery. Dermatomal analysis were not evaluated after surgical procedure. Secondly, the VAS scoring system was used to assess the patients' pain levels. Although the evaluation is done by a single author, individual variability in pain reactions in visual evaluation should be considered [17]. Thirdly, testicular pain, reported to persist postoperatively [18], was not evaluated. Evaluation of the nature of pain and the possibility of the plane block being outside the area of interest may also be the cause of the pain. Additionally; the study's sample size was based on the 2nd hour VAS score. A larger number of patients might be required for analgesic consumption and side effects associated with the block procedure. Finally, although effective analgesic modalities were applied in accordance with the fundementals of ERAS protocols, our study did not evaluate patient satisfaction or cost-effectiveness of TFPB during hospitalization.

To conclude, this study has demonstrated that when compared to surgical site infiltration, which is safer and available, TFPB leads to more effective analgesia in patients undergoing varicocelectomy surgery and may be an important part of multimodal analgesia in these patients.

## Data Availability

The datasets used and analyzed during the current study are available from the corresponding author on reasonable request. Erkan Cem Celik e-mail:drerkancem@yahoo.com.

## Abbreviations

IVC: Inferior vena cava
TFPB: Transversalis fascia plane block
ECG: Electrocardiography
NIBP: Non-invasive blood pressure
SpO_2_: Oxygen saturation
VAS: Visual analog scale
ERAS: Accelerated recovery after surgery
